# Chromosomal Instability in Various Generations of Human Mesenchymal Stem Cells Following the Therapeutic Radiation

**DOI:** 10.1155/2023/9991656

**Published:** 2023-08-29

**Authors:** Majid Sadeghi Moghadam, Hosein Azimian, Jalil Tavakol Afshari, Mohammad Taghi Bahreyni Toossi, Najmeh Kaffash Farkhad, Seyed Hamid Aghaee-Bakhtiari

**Affiliations:** ^1^Department of Medical Physics, Faculty of Medicine, Mashhad University of Medical Sciences, Mashhad, Iran; ^2^Immunology Research Center, Department of Immunology, Faculty of Medicine, Mashhad University of Medical Sciences, Mashhad, Iran; ^3^Department of Medical Biotechnology, Faculty of Medicine, Mashhad University of Medical Sciences, Mashhad, Iran

## Abstract

**Background:**

Radiotherapy is a crucial treatment for most malignancies. However, it can cause several side effects, including the development of secondary malignancies due to radiation-induced genomic instability (RIGI). The aim of this study was to evaluate genomic instability in human mesenchymal stem cells (hMSCs) at different X-ray radiation doses. Additionally, the study aimed to examine the relative expression of certain genes involved in DNA repair, proto-oncogenes, and tumor suppressor genes.

**Methods:**

After extracting, characterizing, and expanding hMSCs, they were exposed to X-ray beams at doses of 0, 0.5, 2, and 6 Gy. Nuclear alterations were evaluated through the cytokinesis-block micronucleus (CBMN) assay at 2, 10, and 15 days postirradiation. The expressions of BRCA1, BRCA2, TP53, Bax, Bcl2, and KRAS genes were analyzed 48 hr after irradiation to evaluate genomic responses to different radiation doses.

**Results:**

The mean incidence of micronuclei, nucleoplasmic bridges, and nuclear buds was 4.8 ± 1.6, 47.6 ± 6, and 18 ± 2.6, respectively, in the nonirradiated group 48 hr after the fourth passage, per 1,000 binucleated cells. The incidence of micronuclei in groups exposed to 0.5, 2, and 6 Gy of radiation was 14.3 ± 4.9, 32.3 ± 6.5, and 55 ± 9.1, respectively, 48 hr after irradiation. The expression levels of the BRCA2, Bax, TP53, and KRAS genes significantly increased after exposure to 6 Gy radiation compared to the control groups. However, there was no significant increase in BRCA1 and Bcl2 gene expression in our study.

**Conclusion:**

This study demonstrated significant nuclear alterations in the 10 days postirradiation due to the RIGIs that they inherited from their irradiated ancestral cells. While chromosomal instability is a prevalent event in malignant cells, so it seems necessary to optimize radiotherapy treatment protocols for tissues that contain stem cells, especially with IMRT, which delivers a low dose to a larger volume of tissues.

## 1. Introduction

Human mesenchymal stem cells (hMSCs), also known as multipotent stromal cells, are present in several tissues such as bone marrow, adipose tissue, connective tissue, cartilage, umbilical cord, and placenta. MSCs play a crucial role in tissue plasticity, wound healing, and the regulation of inflammatory tissue responses [1–4]. They have the ability to differentiate into various specialized cells, such as osteocytes, adipocytes, and chondrocytes. They also have a rich history in treating various diseases and have numerous applications in regenerative medicine [5–7]. MSCs accompany individuals from birth to death, proliferating and differentiating based on tissue needs. Therefore, MSCs and their progeny cells have a prolonged period in the body regarding genomic instability. Ionizing radiation (IR) from diagnostic and therapeutic procedures can have various radiobiological effects on tissues and cells. Direct radiation damage to DNA, as well as aberrations caused by free radicals, activate diverse signaling pathways, including apoptosis, cell cycle arrest, and DNA repair processes.

If DNA damage is not properly and effectively repaired, it can lead to genomic instability, apoptosis, mutation, and other long-term radiation effects. The most crucial late effect of IR is the development of radiation-induced secondary malignancies (RISM). More than 50% of cancer patients receive radiotherapy as a component of their treatment plan. Approximately 1%–5% of patients may develop RISM within a few years after undergoing radiotherapy [8–10]. Radiation-induced genomic instability (RIGI) seems to be the most critical cause of RISM [11].

Although the population of MSCs decreases with age, hMSCs possess long-term repopulation potential (LTRP). This indicates that their genomic integrity remains substantial over a long time window [12].

Genomic instability encompasses a broad spectrum of chromosomal abnormalities such as deletions, translocations, rings, and dicentric, as well as genetic and epigenetic damage. Thus, genomic instability is a broad target for ionizing radiation. In various studies, researchers have used metaphase analysis, cytokinesis-block micronucleus (CBMN) analysis, and gene expression tests to investigate genomic instability [13, 14]. Chromosomal aberrations, including the micronucleus (MN), nucleoplasmic bridge (NPB), and nucleus buds (NBUD), can be evaluated using the CBMN assay [15].

We hypothesized that irradiation of hMSCs leads to genomic instability in the next generations of irradiated cells. We irradiated hMSCs with doses of 0.5, 2, and 6 Gy and evaluated nuclear alterations at 2, 10, and 15 days postirradiation. We compared the results with those of the corresponding control groups in triplicate independent form. To investigate the genomic response of MSCs to radiation, we analyzed the expressions of BRCA1, BRCA2, TP53, Bax, Bcl2, and KRAS genes 48 hr after irradiation.

## 2. Materials and Methods

### 2.1. MSCs Isolation, Preparation, and Characterization

**Figure 1:**
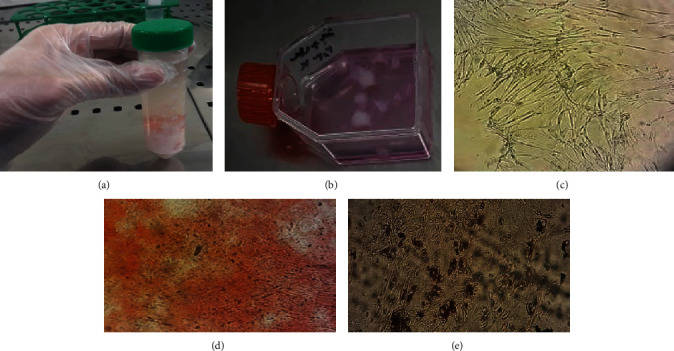
Isolation and extraction steps of MSCs: (a) the Wharton's jelly was carefully separated and cut into small pieces and it was washed several times; (b) extracted cells, as well as Wharton's jelly pieces, were seeded in a culture medium. Wharton's jelly pieces were removed from the flask after 18–21 days; (c) extracted MSCs appeared as spindle-shaped and fibroblast-like cells that adhere to the substrate; (d) osteogenic differentiation of hUCMSc; (e) adipogenic differentiation of MSCs.

hMSCs were extracted from a fresh human umbilical cord. For this aim, umbilical cord was washed with a sterile 0.9% (w/v) saline solution for removing blood cells. The blood vessels were carefully removed by making longitudinal and transverse incisions in the umbilical cord. Wharton's jelly was dissected into 1–3 mm pieces and treated with 0.2% collagenase/dispase (Boehringer Mannheim GombH, Germany) for 1 hr, followed by treatment with trypsin EDTA (Gibco, USA) for 30 min at 37°C with agitation. After centrifugation at 1,500 rpm for 5 min and removal of the supernatant, hMSCs were seeded in 25 cm^2^ flasks (SLP, South Korea) and maintained in a culture medium consisting of *α*-minimum essential medium (*α*-MEM) (Biowest/South America) supplemented with 20% fetal bovine serum (Biowest/South America) and 1% penicillin/streptomycin (Gibco, USA). The cells were then incubated at 37°C with 5% CO_2_. The fibroblast-like adherent cells were digested and replated to expand the cell population. This was done in preparation for creating the master cell bank at the third passage. Then, MSCs were characterized for CD73, CD90, CD34, and CD45, using flow cytometry analysis (Figure 1). Additionally, their ability to differentiate into osteoblasts and adipocytes was evaluated in accordance with the guidelines established by the International Society for Cell & Gene Therapy (ISCT) [16–18].

**Figure 2:**
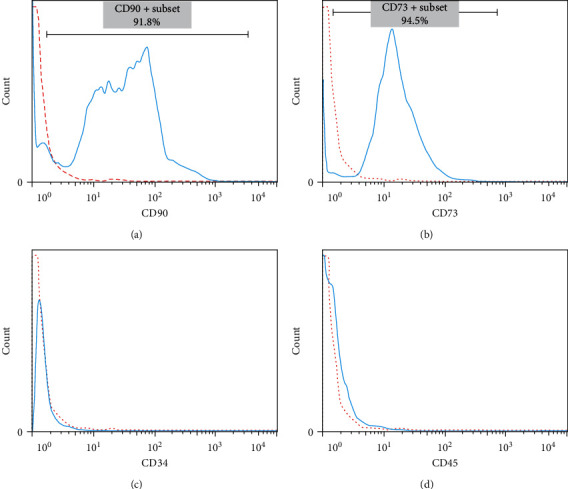
Immunophenotypic characterization of human umbilical cord-derived mesenchymal stem cells was performed using flow cytometry to assess the expression of mesenchymal surface cells marker: (a) CD90; (b) CD73 and hematopoietic stem cell marker; (c) CD34; (d) CD45. (dotted line: unstained control, solid line: a marker of interest).

Osteogenic differentiation of MSCs was evaluated using Alizarin Red S staining (Sigma-Aldrich, UK) and nitroblue tetrazolium/5-Bromo-4-chloro-3-indolyl phosphate (NBT/BCIP, Sigma-Aldrich, UK) as a substrate for alkaline phosphatase (ALP). In addition, we used oil red O staining (Sigma-Aldrich, UK) to examine the adipogenic differentiation potential of MSCs (Figure 2).

### 2.2. Cell Irradiation

We expose the cells with X-ray beam through a 6 MeV linear accelerator (Siemens, Concord, CA, USA) at a dose rate of 2 Gy per minute. A 1.5 cm thick plexiglas plate was used under the flasks to provide the buildup thickness. Cells were cultured 24 hr before irradiation to ensure complete cell's adherence to the substrate.

### 2.3. CBMN Assay

For the CBMN examination, 24 hr prior to the test, the previous culture medium in the flasks was replaced with a fresh medium containing cytochalasin-B (cyt-B) at a final concentration of 4 *μ*g/ml. After treating the cells with cyt-B for 24 hr, they were trypsinized for 3–5 min. The effect of trypsin was neutralized by adding a sufficient amount of *α*-MEM culture medium. The isolated cells were transferred to a falcon tube and centrifuged at 1,600 rpm for 5 min. The supernatant was removed.

The cells were treated and incubated with 1,000 *μ*l of a hypotonic solution of KCl with a molarity of 0.075 for 5 min at room temperature. The process of hypotonic treatment was stopped by adding 500 *μ*l of a cold fixative solution, including 9 : 1 methanol-acetic acid. Tubes containing cells were centrifuged, and the supernatant was removed. At this time, 2 ml of fixative solution was added to the falcon tube, and the cells were evenly distributed by pipetting. Ten minutes were considered the fixation time. The centrifugation process was performed, and the supernatant was discarded. About 300 *μ*l of fresh fixative solution were added to the cells, and the cells were evenly distributed by pipetting.

The cell suspension was uniformly distributed onto the slide. The slides were left to dry completely for approximately 14–16 hr. The slides were stained with 5% Giemsa dye diluted in phosphate-buffered saline (PBS) for 5 min. Stained cells were examined with a light microscope to assess them for micronuclei, nucleoplasmic bridges, and nucleus buds. For more accuracy, the following criteria were taken into consideration [15, 19, 20]:

1. In a binucleated cell, both nuclei should be clearly visible and fully separated without any overlap.
2. The cytoplasmic membrane and the nuclear membrane should be clearly visible.
3. The nuclei had the same color density and pattern.
4. Mononuclear and multinucleated cells, as well as necrotic or apoptotic cells, were excluded from the MN counting.

### 2.4. RNA Extraction and cDNA Synthesis

A Total RNA Extraction Kit (Pars-Tous, Iran) was used to extract RNA following the manufacturer's instructions. The cells were digested using trypsin EDTA (Gibco, USA), washed with PBS, and then centrifuged for 5 min at 1,500 rpm. The supernatant was removed, and 750 *μ*l of RL solution (Total RNA Extraction Kit, Pars-Tous, Iran) was added to the cells. The mixture was then centrifuged for 5 min at room temperature. After centrifugation, 150 *μ*l of chloroform was added to the mixture, which was then vortexed for 15 s and incubated for 3 min at room temperature. The cell solution was transferred to 2 ml microtubes and centrifuged for 12 min at 13,000 rpm. Four thousand microliter of the supernatant was mixed with an equal volume of 70% ethanol and then transferred to a column filter in a new microtube. The mixture was then spun for 1 min. The content on the filter, containing RNA, was washed with 750 *μ*l of PW solution (Pars-Tous, Iran) and centrifuged for 1 min. Finally, the RNA content on the filter was spun for 1 min at 13,000 rpm with 50 *μ*l of DEPC water. The RNA solution was stored at −80°C.

Complementary DNA (cDNA) Synthesis Kit (Pars-Tous, Iran) was used to synthesize cDNA. First, a master mix was prepared by combining 10 *μ*l of buffer mix and 2 *μ*l of mixing enzyme (Pars-Tous, Iran). Twelve microliters of the master mix was added to each microtube containing 8 *μ*l of RNA solution, which contained a total of 1 *μ*g of RNA. Microtubes were vortexed to homogenize the solutions and then transferred to a thermocycler. The temperature and reaction time were adjusted according to the conditions proposed by Pars-Tous Company. Prepared cDNA was stored at a temperature of −20°C.

### 2.5. Gene Expression Experiment

Quantitative real-time polymerase chain reaction (RT-PCR) was used to assess gene expression. RT-PCR reactions were performed in triplicate using a StepOnePlus™ (96-well) Real-Time PCR System (Applied Biosystems) with a total volume of 20 *µ*l. The reaction mixture included 1.5 *µ*l of cDNA, 10 *µ*l of SYBR® Green Real-Time PCR Master Mix, 0.4 *µ*l of ROX reference dye (Takara, Japan), 6.1 *µ*l of dH_2_O, and 300 nM of both forward and reverse primers.

The beta-2 microglobulin (B2M) gene was used as the housekeeping gene to normalize the quantity of target genes. StepOneTM software version 2.3 (Applied Biosystems) is used for data analysis after every run. For the final calculation of relative quantity (RQ), we calculated the ratio of the normalized quantity of the treated samples to the normalized quantity of the control sample.

The BRCA1, BRCA2, Bcl2, Bax, TP53, and KRAS genes were considered as target genes. The primers for these genes were designed and synthesized as exon junctions with a length of 18–30 bp, 40%–60% CG content, and a melting temperature of 55–60°C. For this survey, the primers were provided by Metabion (Martinsried, Germany).

### 2.6. Statistical Analysis

Prism (version 6.0) for Windows (GraphPad Software, San Diego, CA, USA) and Excel (Microsoft Office 2016) were utilized for data analysis. The normality of the data was assessed using both the Kolmogorov–Smirnov and Shapiro–Wilk tests. Mean comparisons were conducted using the *t*-test and one-way ANOVA.

## 3. Results

### 3.1. Cells Extraction and Characterization

The current study involved the extraction and cultivation of hMSCS from the Wharton's jelly region of the umbilical cord. Once the primary cells reached 80% confluence, they were subcultured to establish a cell bank. Extracted cells had an average doubling time of approximately 20 ± 4 hr. The cells were spindle-shaped, fibroblast-like, and attached to the substrate. After specific cell staining, the cells demonstrated the ability to differentiate into osteoblasts and adipocytes (Figure 1).

Practically, all cells demonstrated high expression of MSCs markers, including CD73 and CD90, while lacking expression of hematopoietic stem cell markers CD34 and CD45 (Figure 2). The extracted cells exhibited a fibroblast-like morphology and expressed high levels of MSCs markers that indicated their mesenchymal nature.

### 3.2. Micronucleus Assessment

**Figure 3:**
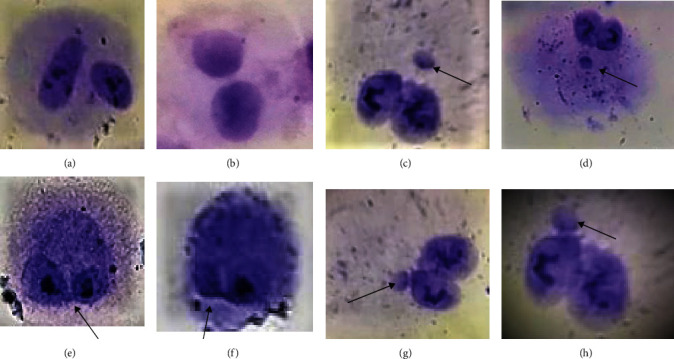
Nuclear alterations in the CBMN assay: (a and b) binucleated cell; (c and d) binucleated cell with MN; (e and f) binucleated cell contained NPB; (g and h) binucleated cell with NBUD.

We investigated nuclear alterations in MSCs before and postirradiation. After extracting and characterizing the cells, the master cell bank was developed. In the fourth passage, MSCs in the zero, half, two, and six radiation groups were irradiated in separate flasks. All groups were independently examined in triplicate. Nuclear alterations in binucleated cells (BNs) were analyzed using the CBMN assay at 2, 10, and 15 days postirradiation. BNs were examined for MNs, NPBs, and NBUDs (Figure 3).

**Figure 4:**
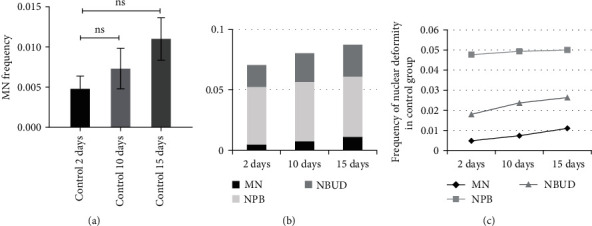
Chromosomal aberration assessment in the control group of MSCs. The experiment was conducted with three independent replications using a standard culture medium: (a) MN frequency (mean ± SD) per 1,000 binucleated cells was evaluated at 2, 10, and 15 days after the fourth passage. Mean comparison conducted via one-way ANOVA test (ns, not significant); (b) cumulative diagram of the mean nuclear alterations, including MNs, NPBs, and NBUDs, in binucleated cells; (c) mean nuclear alterations in mesenchymal stem cells over the time.

The control group was examined using the CBMN assay at 2, 10, and 15 days after the fourth passage. The mean incidence of MNs, NPBs, and NBUDs for 1,000 BNs was 4.8 ± 1.6, 47.6 ± 6, and 18 ± 2.6, respectively, 48 hr after the fourth passage in the control group. With the increase in the number of cell passages in MSCs, there was a slight increase in the incidence of MN, although it was not statistically significant (Figure 4(a)).

Furthermore, the MSCs in the control group underwent the MN test on the 10th and 15th days after the fourth passage.  Figure 4(b) illustrates that the level of chromosomal damage rises with an increase in the number of cell passages. NPB appeared to have a greater contribution than the other markers. Also, we demonstrated that the MNs, NPBs, and NBUDs frequencies rise in a linear fashion with varying slopes as the number of cell passages increased (Figure 4(c)).

**Figure 5:**
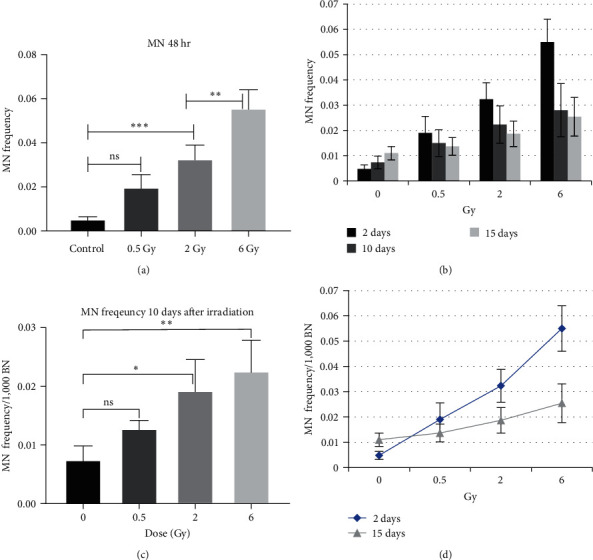
MN frequency (per 1,000 binucleated cells) and mean comparison analysis through the one-way ANOVA test at various radiation doses. Results represent the mean ± SD of three independent replications of each radiation group: (a) MN incidence rate 2 days after irradiation; (b) the effect of dose on the MN incidence; (c) MN incidence rate at 10 days after irradiation; (d) MN incidence variations 2 and 15 days postirradiation (ns, not significant,  ^*∗*^*P*-value < 0.05,  ^*∗∗*^*P*-value < 0.002,  ^*∗∗∗*^*P*-value < 0.0002).

The incidence of MN was investigated in various radiation groups, including those exposed to 0.5, 2, and 6 Gy at 2, 10, and 15 days after irradiation. Each radiation group was independently replicated three times. The chromosomal damage in each radiation group was compared to a corresponding control group that was maintained under identical culture and passage conditions. This allowed us to determine that any difference in nuclear alterations between the radiation and control groups was attributable to the genotoxic effects of the radiation (Figure 5).

**Figure 6:**
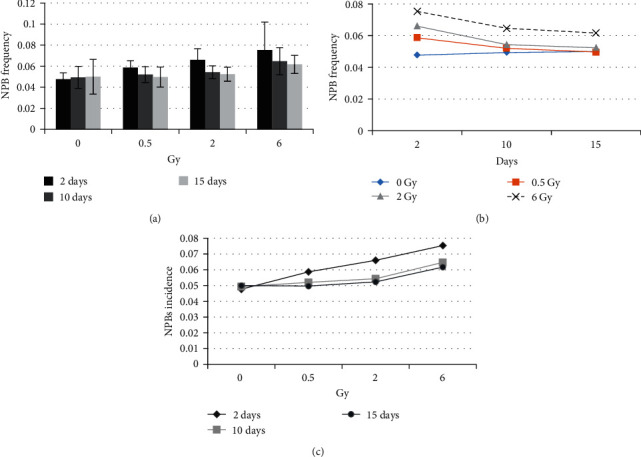
NPB frequency (per 1,000 binucleated cells), all radiation groups are cultivated in standard culture in independent triplicate form: (a) NPB incidences for various radiation doses (mean ± SD); (b) mean alterations in NPB incidence over the time; (c) mean alterations in NPBs incidence with radiation dose.

NPB is an indicator of genomic instability, which may be due to telomeric fusion and anaphase bridges between daughter cells during the cytokinesis stage of mitotic division [13]. In this study, we investigated the occurrence rate of NPBs at different radiation doses and times (Figure 6).

**Figure 7:**
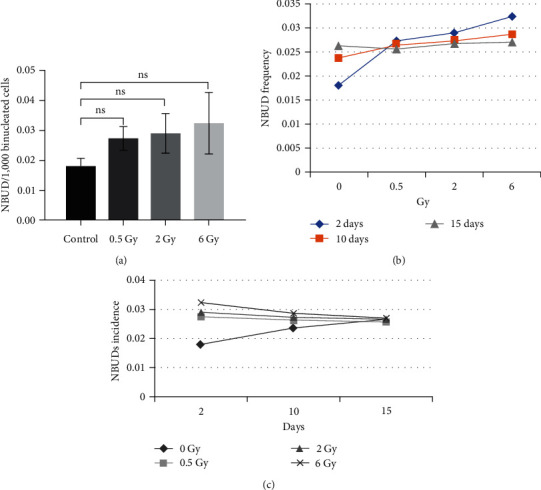
The incidence of NBUDs per 1,000 binucleated cells: (a) NBUDs incidence in different radiation groups, 2 days postirradiation. All groups are cultivated in a standard culture in triplicate independent form. Results are presented as mean ± SD (ns, not significant); (b) mean NBUDs variation with the radiation dose; (c) mean NBUDs alteration in subsequent cell generations.

Some researchers attribute the occurrence of NBUDs to genomic proliferation, while others suggest that they may result from the breakdown of NPBs [19]. In any case, the incidence of NBUDs indicates that the genomic integrity of the cell has been impaired. The incidence of NBUDs for various radiation doses and times is presented in Figure 7.

### 3.3. Gene Expression

**Figure 8:**
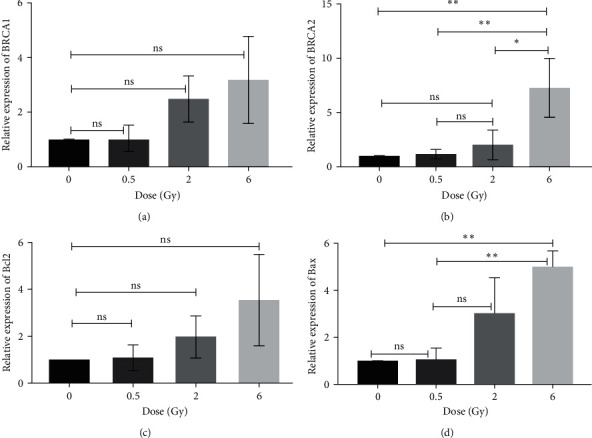
Relative gene expression in human mesenchymal stem cells for various radiation doses 48 hr postirradiation. All experiments were examined in independent triplicate form and the results are represented as mean ± SD: (a) BRCA1; (b) BRCA2; (c) Bcl2; (d) Bax (ns, not significant,  ^*∗*^*P*-value < 0.05,  ^*∗∗*^*P*-value < 0.002).

We examined the expression of several genes, including BRCA1, BRCA2, Bcl2, Bax, Tp53, and KRAS, in hMSCs for the control group and those exposed to half, two, and six radiation doses. Our aim was to investigate the response of genes involved in DNA damage repair (DDR), apoptosis, cell cycle control, and cell proliferation. Our findings demonstrate an increase in BRCA1, BRCA2, Bcl2, and Bax gene expressions 48 hr after irradiation with increasing radiation doses. Although Tukey's multiple comparison tests were not significant in the one-way ANOVA test for BRCA1 and Bcl2 in various radiation dose groups (Figure 8).

Contrary to Bcl2 and BRCA1, the expression values of the Bax and BRCA2 genes in the 6 Gy dose compared to the half and sham groups indicated a significant increase (Figures 8(b) and 8(d)).

**Figure 9:**
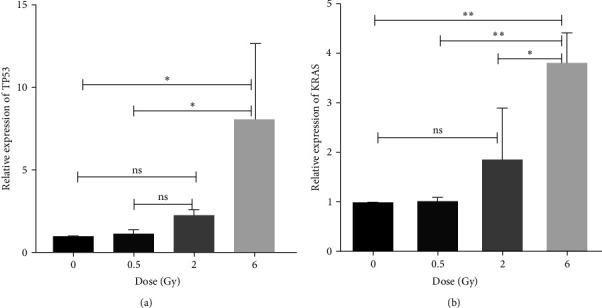
Relative gene expression in human mesenchymal stem cells for various radiation doses 48 hr postirradiation. All experiments were examined in independent triplicate cell cultivated groups and results are represented as mean ± SD: (a) TP53; (b) KRAS (ns, not significant,  ^*∗*^*P*-value < 0.05,  ^*∗∗*^*P*-value < 0.002).

TP53 as a substantial tumor suppressor showed a significant increase in the 6 Gy dose compared to half gray and sham groups (Figure 9(a)). Also, this survey demonstrated upregulation of KRAS gene, 48 hr after irradiation (Figure 9(b)).

## 4. Discussion

Radiation effects on various cell lines have been investigated in numerous studies. Most of these studies have been conducted on genetically manipulated immortalized cell lines. However, these findings may not necessarily reflect normal cellular responses [21, 22]. In this study, normal hMSCs were used to investigate the long-term radiation effects.

We extracted hMSCs from the umbilical cord using antiseptic techniques, following the protocols established by the ISCT for extracting and characterizing stem cells [17, 18]. The high purity of the extracted MSCs was demonstrated by the expression of over 90% for MSC markers (CD73 and CD90) and low expression of hematopoietic markers (CD34 and CD45), as well as the uniform morphology of cells (Figure 2).

Examination of the CBMN test for MSCs in the sham group indicated increase in chromosomal instability during cell passages and the development of the cell bank. In this study, the numbers of MN, NPB, and NBUD increased over time, specifically on the 10th and 15th days after the fourth passage in the sham group. However, these increases did not reach statistical significance. These elevations in chromosomal aberrations may be due to cellular aging and a decrease in cellular repair capacity (Figure 4). However, millions of MSCs are required for each patient in cell therapy and regenerative medicine. So, even minor genomic instability in these cells can lead to future complications for the patient, such as the development of malignancy. It appears that the conditions for developing cell banks should be optimized in terms of maintenance, micronutrients, and cell passages. Furthermore, prior to clinical application, the level of genomic instability in the cells must be evaluated.

In this study, the values of MN and NBUD for the control group were consistent with those determined by other researchers [19, 23]. The MN values counted are lower than the mean MN for the XP4PA cell line reported in Cornelio et al.'s [19] study. This finding may indicate that MSCs have more robust repair mechanisms compared to the XP4PA cell line.

The mean frequency of MN in the different radiation groups is presented in Figure 5. Curve fitting analysis of the point graph indicates that the incidence of MN increases exponentially with radiation dose (*R*^2^ = 0.983), 48 hr after irradiation. Doses greater than 2 Gy resulted in a significant increase in the number of MN compared to the sham groups (Figure 5(a)). This finding suggests that increasing the radiation dose beyond 2 Gy results in higher tissue ionization and oxidative stress. Consequently, the intensity of DNA damage exceeds the cell's repair capacity.

MN and NPB incidences are often considered fatal damages. Cells with these damages gradually removed from the cell population through apoptosis and mitotic death. So, the significant occurrence of micronuclei 10 days after radiation exposure was likely due to the RIGI that they inherited from their irradiated ancestral cells. Based on a doubling time of 16–24 hr, the cells on the 10th day can be considered as the 15th to 10th cell generations. (Figure 5(c)). An increase in the incidence of MN proportional to the radiation dose was observed in irradiated cells that underwent the CBMN test 15 days after exposure. However, this increase was not statistically significant (Figure 5(d)). These findings are consistent with the results of the study conducted by Huumonen et al. [22].

NPB is a chromosomal aberration marker that arises from telomeric fusion, as well as anaphase bridges between two daughter cells during the cytokinesis stage of mitotic division [13]. In our study, the NPB variable increased with the rising dose at different times postirradiation. Although Tukey's multiple comparison tests did not yield significant results in the one-way ANOVA test for NPBs in various radiation dose groups (*P*-value > 0.05) (Figure 6(a)). For different doses of radiation, the highest incidence of NPB was observed on the 2nd day after radiation. The incidence of NPB in the subsequent cell generations (on the 10th and 15th days) decreased compared to the 2nd day (Figure 6(c)). This may be attributed to the death of cells with genomic damage. However, cells on the 10th and 15th days had not received radiation themselves but they had inherited chromosomal damage from their irradiated ancestors.

NBUDs are morphologically similar to micronuclei, except they are attached to the nucleus. They are markers of DNA replications that remove from the cell nucleus and also result from NPB breakage during the cytokinesis process.

The incidence rate of NBUDs increased linearly with increasing doses in various radiation exposure groups, as shown in Figure 7(b). However, ANOVA analysis indicated that there was no significant difference in the mean incidence of NBUDs between the radiation groups 2 days postirradiation (*P*-value > 0.05). The highest incidence of NBUDs was observed on the 2nd day after radiation for different doses of radiation. The number of NBUDs decreased gradually over a period of 10–15 days after irradiation (Figure 7(c)). This may be attributed to the death of cells with genomic damage.

Ionizing radiation can cause various types of damage to the DNA structure, including single-stranded breaks (SSBs) and double-stranded breaks (DSBs). SSB repair pathways are typically error-free due to the presence of sister chromatids. However, DSB repair can occur through either the precise repair pathway of homologous recombination (HR) or the error-prone nonhomologous end joining (NHEJ) pathway. The DSB repair through the HR is mediated by BRCA1, BRCA2, and RAD51 [24].

Our study demonstrated a linear increase in BRCA1 expression as the radiation dose increased, but this increase was not statistically significant (Figure 8(a)). Upregulation of the BRCA2 gene was observed with an increasing radiation dose at 48 hr. This increase was statistically significant for a dose of 6 Gy compared to the control group, as well as for doses of 0.5 and 2 Gy (Figure 8(b)). BRCA family upregulation may indicate activation of HR repair pathways, as well as cell cycle arrest in the G2M and S phases. Cell cycle arrest provides an opportunity to repair DNA damage [25]. Today, the crucial role of the BRCA1 and BRCA2 genes in the precise repair of genomic and DNA damage has been largely clarified [25, 26].

The Bcl2 gene acts as antiapoptotic and prosurvival mediator [27, 28]. The Bcl2 protein reduces the permeability of the mitochondrial membrane to the proapoptotic protein cytochrome C [28, 29]. Our findings demonstrate that irradiated MSCs exhibit a linear increase in Bcl2 levels within 48 hr after irradiation. However, the expression level did not show a statistically significant difference among the different radiation groups (Figure 8(c)). These results are consistent with those reported by Huumonen et al. [22].

The Bax gene is a proapoptotic member of the Bcl2 family that activates the internal pathway of apoptosis by increasing the permeability of the mitochondrial membrane [30, 31]. We observed that the expression of the Bax gene increased proportionally with the radiation dose. This increase was statistically significant for the dose of 6 Gy compared to the control and 0.5 Gy radiation dose groups (Figure 8(d)). High expression of the Bax gene indicates significant activation of the internal apoptosis pathway, resulting in the elimination of damaged cells.

Cell irradiation induces ATM phosphorylation and is followed by upregulation of TP53. The TP53 gene causes an increase in the expression of Bax family genes, thereby activating the internal pathway of apoptosis. It can also lead to an increase in ligands and cell death receptors, activating the external pathway of apoptosis [32]. Other studies have shown that TP53 can induce cell cycle arrest in the G1 phase through the P21/Waf1 pathway, as well as in the G2/M cell cycle arrest through the GADD45 pathway. Cell cycle arrest provides an opportunity to repair DNA damage [33, 34]. In our study, we observed an increase in Tp53 gene expression with increasing radiation dose at 48 hr postirradiation. This increase was statistically significant for the 6 Gy dose group compared to both the control and 0.5 Gy groups (Figure 9(a)). TP53 upregulation acts as a robust signaling modulator for repair, as well as the removal of cells with genomic damage through the aforementioned pathways. This result is consistent with the survey conducted by Nicolay et al. [35].

The results showed that the KRAS gene expression increases exponentially with radiation dose (*R*^2^ = 0.898). The rate of expression for doses of 2 and 6 Gy was significantly higher than the sham and 0.5 Gy radiation groups (*P*-value < 0.002) (Figure 9(b)).

KRAS is a member of the RAS family, a group of protein factors that promote cell survival and proliferation in response to an increase in the epidermal growth factor receptor (EGFR) ligand. This signaling pathway can be activated by changes in the biochemistry of the cell's microenvironment, which may occur due to exposure to ionizing radiation and tissue damage [36, 37]. It seems that the high expression level of KRAS acts as a contributory factor for the survival and proliferation in irradiated MSCs.

## 5. Conclusion

hMSCs are undifferentiated cells that possess the potential for long-term repopulation. However, exposure to radiation can induce genomic instability in these cells. This study demonstrated an increase in the expression of BRCA2, TP53, and Bax 2 days after irradiation, leading to the activation of various pathways involved in DNA repair and apoptosis. Nevertheless, chromosomal aberration increased with the dose, in irradiated cells 2 days postirradiation, as well as in the next generations of hMSCs due to inherited RIGI from ancestral irradiated hMSCs. Chromosomal instability is a common characteristic observed in malignant cells. Therefore, it is possible that genomic instability in hMSCs may lead to the development of secondary malignancies, including various types of sarcoma, in the years following radiotherapy treatment. So, it is necessary to optimize radiotherapy treatment protocols for tissues that contain stem cells, especially with IMRT. This technique delivers a low dose to a larger volume of tissues.

## Data Availability

All data generated or analyzed during this study are included in this published article.

## Abbreviations

hMSCs: Human mesenchymal stem cells
UC-MSCs: Umbilical cord-derived MSCs
TP53: Tumor protein p53
Bcl2: B-cell lymphoma 2
Bax: Bcl-2 associated X-protein
KRAS: Kirsten rat sarcoma virus
IR: Ionizing radiation
RISM: Radiation-induced secondary malignancy
RIGI: Radiation-induced genomic Instability
LTRP: Long-term repopulation potential
CBMN: Cytokinesis-block micronucleus
MN: Micronucleus
NPB: Nucleoplasmic bridge
NBUD: Nucleus bud
ISCT: International Society for Cell & Gene Therapy
ALP: Alkaline phosphatase
Cyt-B: Cytochalasin-B
PBS: Phosphate-buffered saline
RT-PCR: Real-time polymerase chain reaction
B2M: Beta-2 microglobulin
BNs: Binucleated cells
DDR: DNA damage response
ATM: Ataxia-telangiectasia mutated
ɣH2AX: GammaH2AX
Mcl-1: Myeloid cell leukemia
Bfl1: Bcl-2-related gene expressed in fetal liver
Bak: Bcl-2 homologous antagonist/killer
Bok: Bcl-2 related ovarian killer
GADD: Growth arrest and DNA damage-inducible protein.
